# Long-Term Nitric Oxide Exposure Enhances Lung Cancer Cell Migration

**DOI:** 10.1155/2013/186972

**Published:** 2013-07-31

**Authors:** Arpasinee Sanuphan, Preedakorn Chunhacha, Varisa Pongrakhananon, Pithi Chanvorachote

**Affiliations:** Department of Pharmacology and Physiology, Faculty of Pharmaceutical Sciences and Cell-based Drug and Health Product Development Research Unit, Chulalongkorn University, Bangkok, Thailand

## Abstract

Nitric oxide (NO) found in the vicinity of lung cancer cells may play a role in the regulation of cancer cell behaviors. To explore the possible effects of NO on cell motility, human lung cancer cells were exposed to nontoxic concentrations of NO for 0–14 days, and the migratory characteristics of the cells were determined. The present study found that long-term treatment with NO significantly enhanced cell migration in a dose- and time-dependent manner. Furthermore, we found that the increased migratory action was associated with the increased expression of caveolin-1 (Cav-1), which in turn activated the focal adhesion kinase (FAK) and ATP-dependent tyrosine kinase (Akt) pathways. Notably, the NO-treated cells exhibited an increased number of filopodia per cell, as well as an increase in the levels of cell division cycle 42 (Cdc42) protein. Together, these results indicate that extended NO exposure has a novel effect on cell migration through a Cav-1-dependent mechanism, a finding that strengthens our understanding of cancer biology.

## 1. Introduction

The cancer microenvironment has been reported to have a significant impact on cancer cells in many ways [1]. Indeed, in such an active environment, cell signaling molecules as well as mediators including proinflammatory cytokines and reactive species are found to be intensified [2]. Among them, the concentrations of nitric oxide (NO), a reactive nitrogen species synthesized by many cells, such as endothelial, immune, and tumor cells, are found to be dramatically increased in lung cancer environments [3, 4]. Excessive and uncontrolled NO production is associated with the pathogenesis of lung cancer [5]. Additionally, clinical observation has shown that NO levels in the lungs of lung cancer patients were increased in comparison to those of normal subjects [6, 7]. While cytokines have been shown to have significant effects on the behavior of cancer cells within microenvironment, the effects of long-term nitric oxide exposure on lung cancer cell motility remain unknown. 

The ability of cancer cells to migrate is an important hallmark of successful metastasis [8]. The metastasis cascade is a multistep process that consists of five components: local migration and invasion, intravasation, circulation, extravasation, and colony formation at secondary sites [9]. Tumor cells need to be motile to invade tissues; this motility is achieved by changing their cell-cell adhesion properties and by reorganizing their cytoskeletons. These cellular mechanisms are regulated by various signaling molecules, including the Rho family of small GTPases, caveolin-1 (Cav-1), and focal adhesion kinase (FAK) [10, 11]. FAK is activated by an initial autophosphorylation at the Tyr 397 residue, and its activation is essential for the regulation of focal adhesion turnover and cell protrusion [12, 13]. Studies have reported that FAK mediates cells motility through the activation of the downstream Akt signaling pathway [14]. Furthermore, evidence has suggested that Cdc42 overexpression increased cell motility by inducing the formation of filopodia [11, 15, 16]. Recently, caveolin-1 (Cav-1), a 21–24 kDa integral membrane protein, has garnered increasing attention as its role in the regulation of cancer cell behaviors has been revealed [17–26]. Increased Cav-1 expression was shown to be associated with enhanced progression of prostate, colon, and breast cancers [26, 27]. Likewise, elevated Cav-1 expression was associated with an increased metastasis capacity and poor survival in lung cancer patients [26, 28]. We investigated the role of long-term exposure to nontoxic doses of NO on lung carcinoma cell motility and examined the possible underlying mechanisms using pharmacological approaches. The findings of the present study aid in the better understanding of this microenvironment-related mediator and may help in the development of novel anticancer strategies. 

## 2. Materials and Methods

### 2.1. Cells and Reagents

Human non-small-cell lung cancer cells (NCI-H460) were obtained from the American Type Culture Collection ((ATCC) Manassas, VA, USA). Cells were cultured in RPMI 1640 medium supplemented with 5% fetal bovine serum, 2 mM L-glutamine, 100 IU/mL penicillin, and 100 *μ*g/mL streptomycin (Gibco, MD, USA) in a humidified atmosphere of 5% CO_2_ at 37°C. For long-term exposure experiments, cells were cultured in medium containing NO donor dipropylenetriamine (DPTA) NONOate (0, 5, and 10 *μ*M) for 7 and 14 days, respectively. The culturing medium was replaced by medium containing the freshly prepared NO donor every 2 days. The NO donor dipropylenetriamine (DPTA) NONOate was purchased from Santa Cruz Biotechnology (Santa Cruz, CA, USA). The 3-(4,5-dimethylthiazol-2-yl)-2,5-diphenyltetrazolium bromide (MTT), Hoechst 33342, phalloidin tetramethylrhodamine B isothiocyanate, sulforhodamine B (SRB), bovine serum albumin (BSA), and dimethylsulfoxide (DMSO) were purchased from Sigma Chemical, Inc. (St. Louis, MO, USA). Antibodies for phosphorylated Akt (S473), Akt, phosphorylated FAK (Y397), FAK, Cdc42, Cav-1, *β*-actin, and peroxidase-conjugated secondary antibodies were obtained from Cell Signaling (Danvers, MA, USA). Lipofectamine 2000 and PrestoBlue were obtained from Invitrogen (Carlsbad, CA, USA).

### 2.2. Plasmids and Transfection

The Cav-1 expression plasmid was obtained from the American Type Culture Collection ((ATCC) Manassas, VA, USA), and the Cav-1 short hairpin knockdown plasmid (shRNA-Cav-1) was obtained from Santa Cruz Biotechnology (Santa Cruz, CA, USA). Stable transfection of cells with the Cav-1 expression plasmid or the Cav-1 knockdown plasmid was achieved by culturing the cells until they reached approximately 60% confluence. Then, 15 *μ*L lipofectamine 2000 reagent and 2 *μ*g Cav-1 expression plasmid, shRNA-Cav-1, or control plasmid were used to transfect the cells in the absence of serum. After 12 h, the medium was replaced with fresh culture medium containing 5% FBS. Approximately 36 h after the beginning of the transfection, the cells were digested with 0.03% trypsin, and the cell suspensions were plated in 75 mL culture flasks and cultured for 20 to 30 days with antibiotic selection. The stable transfectants were pooled, and the expression of the Cav-1 protein in the transfectants was confirmed by Western blotting. The cells were cultured in antibiotic-free RPMI 1640 medium for at least two passages before experiments were performed.

### 2.3. Cytotoxicity Assay

Cell viability was determined using the MTT assay. After treatment, the cells were treated with MTT (5.0 mg/mL in PBS) and incubated for 4 h at 37°C. Then, the MTT solution was removed, and 100 *μ*L DMSO was added to dissolve the formazan crystal. The intensity of the formazan product was measured at 570 nm using a microplate reader (Anthros, Durham, NC, USA). The percentage of cell viability was calculated using the following formula:

(1) $$\begin{array}{c} \text{cell}\;\text{viability}\;\left(\%\right)\;=\;\frac{\left(\text{A}570\;\text{of}\;\text{treatment}\times 100\right)}{\text{A}570\;\text{of}\;\text{control}}. \end{array}$$

### 2.4. Cell Proliferation Assay

Cells were exposed to the NO donor at various concentrations and were subjected to the cell proliferation assay for 0, 24, and 48 h. Cells were seeded at a density of 5 × 10^3^ cells/well in a 96-well plate. Cell proliferation was determined through incubation with PrestoBlue at a 1 : 10 dilution for 1 h, and the fluorescence intensity of the resazurin product (Resorufin) was measured at 530 nm (excitation wavelength) and 590 nm (emission wavelength).

### 2.5. Cell Migration

Cell migration was determined using a wound-healing assay. Cells were grown to a confluent monolayer in a 24-well plate, and then a scrape was made down the center of the well using a P200 micropipette tip. The well was then rinsed with phosphate-buffered saline (PBS) and replaced with RPMI medium. At the indicated times (0, 12, and 24 h), the wound spaces were imaged under a phase-contrast microscope (10X) (Olympus IX51 with DP70), and the wound spaces were measured on the image field at four points per field. Relative cell migration was calculated by dividing the percentage change in the wound space of the treated cells by that of the control cells in each experiment.

### 2.6. Invasion Assay

The invasion assay was performed using a Boyden chamber precoated with 50 *μ*L 0.5% Matrigel (BD Biosciences, MA, USA) on the upper surface of the chamber [29]. Cells were seeded at a density of 3 × 10^4^ cells/well in the upper chamber in serum-free conditions. RPMI medium containing 10% FBS was added to the lower chamber of the unit. After incubation for 24 h at 37°C, the cells in the upper chamber were removed with a cotton swab and the cells in the bottom unit were fixed with cold absolute methanol for 10 min and stained with 10 *μ*g/mL Hoechst 33342 for 10 min. The cells were then visualized and scored under a fluorescence microscope (Olympus IX51 with DP70).

### 2.7. Morphological Characteristics of Cancer Cells

Cell morphology was investigated using phalloidin-rhodamine and sulforhodamine B staining assays. After NO exposure, the cells were fixed with 4% paraformaldehyde in PBS for 10 min at 37°C, permeabilized with 0.1% Triton-X100 in PBS for 4 min, rinsed with PBS, and then blocked with 0.2% BSA for 30 min. The cells were then incubated with either a 1 : 100 dilution of phalloidin-rhodamine in PBS or 0.4% sulforhodamine B in 1% acetic acid for 15 min; the cells were then rinsed 3 times with PBS and mounted with 50% glycerol. The cell morphology was imaged using a fluorescence microscope (Olympus IX51 with DP70).

### 2.8. Western Blotting

After specific treatment, cells were incubated with lysis buffer containing 20 mM Tris·HCl (pH 7.5), 1% Triton X-100, 150 mM sodium chloride, 10% glycerol, 1 mM sodium orthovanadate, 50 mM sodium fluoride, 100 mM phenylmethylsulfonyl fluoride, and protease inhibitor cocktail (Roche Molecular Biochemicals) for 30 min on ice. The cell lysates were collected and determined for protein content using the BCA protein assay kit (Pierce Biotechnology, Rockford, IL, USA). Equal amounts of protein from each sample (40 *μ*g) were denatured by heating at 95°C for 5 min with Laemmli loading buffer and loaded onto 10% SDS-polyacrylamide gel electrophoresis. After separation, proteins were transferred onto 0.45 *μ*m nitrocellulose membranes (Bio-Rad). The transferred membranes were blocked in 5% nonfat dry milk in TBST (25 mM Tris-HCl (pH 7.5), 125 mM NaCl, and 0.05% Tween 20) for 30 min and incubated with the appropriate primary antibodies overnight at 4°C. Membranes were washed three times with TBST for 10 min and incubated with horseradish peroxidase- (HRP-) labeled secondary antibodies for 1 h at room temperature. The immune complexes were detected by chemiluminescence (Supersignal West Pico; Pierce, Rockford, IL, USA) and quantified using analyst/PC densitometry software (Bio-Rad).

### 2.9. Statistical Analysis

The mean data from independent experiments were normalized to the results of the control cells. The values are presented as the mean ± standard deviation (SD) from three or more independent experiments and were analyzed using one-way ANOVA with a post-hoc test (Tukey's test) at a significance level of *P* < 0.05 using SPSS version 16.0. 

## 3. Results

### 3.1. Effect of NO Donor on the Viability of the Human Lung Cancer H460 Cell Line

**Figure 1:**
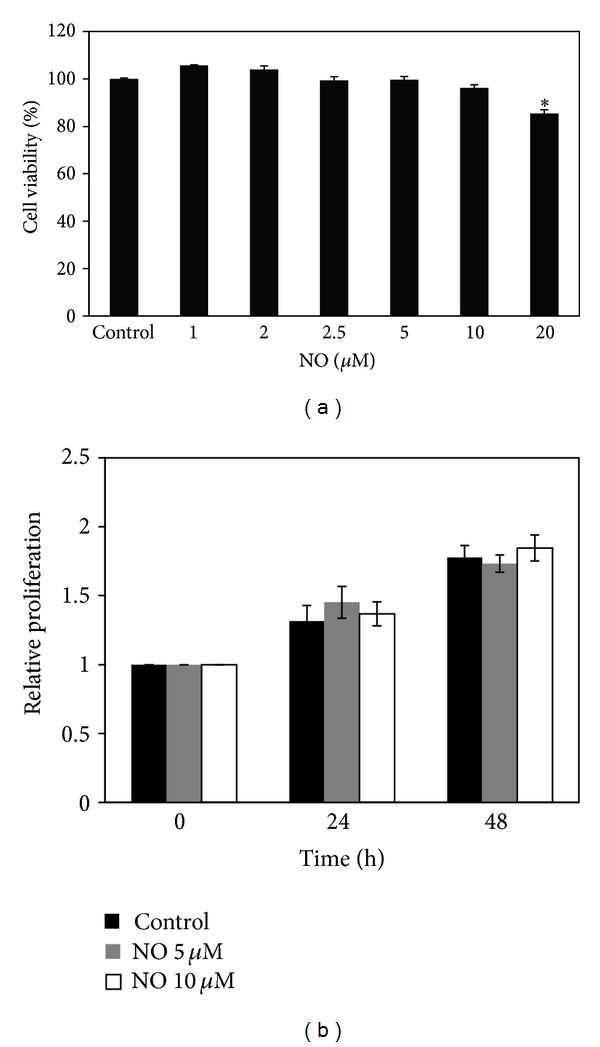
Effect of NO donor on cytotoxicity in lung carcinoma H460 cells. (a) Effect of DPTA NONOate on H460 cell viability. H460 cells were treated with various concentrations (0–20 *μ*M) of DPTA NONOate for 24 h. The cell viability was analyzed using the MTT assay. (b) Proliferative effect of DPTA NONOate on H460 cells. Cell proliferation for 24 and 48 h was determined using PrestoBlue. The data are the mean ± SD (*n* = 3). *P < 0.05 versus the nontreated control.

We first characterized the effects of NO donor on the viability of the human lung cancer H460 cell line. The H460 cells were cultured in the presence and absence of DPTA NONOate (1–20 *μ*M), a slow-releasing NO donor compound, for 24 h, and cell viability was determined.  Figure 1(a) shows that when cells were treated with the NO donor, at concentrations ranging 1–10 *μ*M, neither cytotoxicity nor proliferative effects were observed in the cells. A significant decrease in viability was first detected in cells treated with 20 *μ*M DPTA NONOate; however, approximately 90% of the cells still remained viable. Accordingly, our results indicated that at the indicated doses, the NO donor did not cause a significant effect on cell viability up to 72 h of NO exposure (data not shown). To investigate the effect of long-term NO treatment on cell proliferation, H460 cells were cultured in their optimal conditions supplemented with 5 or 10 *μ*M NO donor, and their proliferative behavior was evaluated using PrestoBlue. As Figure 1(b) indicates, the NO-treated cells exhibited no significant changes in cell proliferation during the test period. 

### 3.2. Long-Term NO Exposure Potentiates Migration and Invasion of H460 Cells

**Figure 2:**
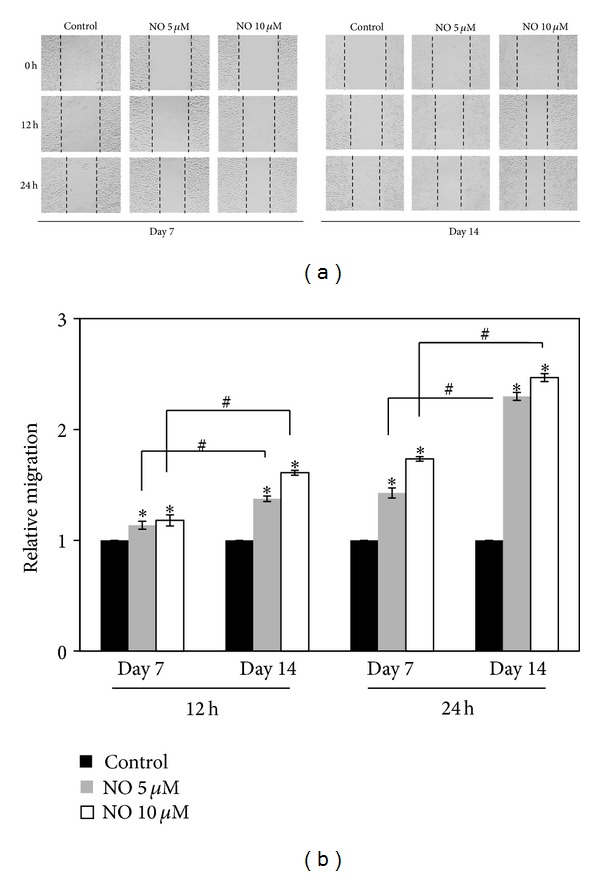
Effect of nitric oxide exposure on H460 cell migration. (a) Cells were exposed to NO donor at various concentrations for 7 or 14 days and subjected to a migration assay. Phase-contrast images were captured at 0, 12, and 24 h. (b) The relative cell migration was determined by comparing the relative change in wound space to the control cells. The data are the mean ± SD (*n* = 3). *P < 0.05 versus the control cells, ^#^ *P* < 0.05 versus NO-treated cells at 7 days.

To investigate the effect of NO on cell migration, we performed scratch wound-healing assays. Cells were exposed to NO for 7 or 14 days and were subjected to the migration assay for 12 and 24 h. Figures 2(a) and 2(b) show that long-term treatment with the NO donor significantly enhanced the motility of the cells in dose- and time-dependent manners as compared with the H460 control cells. Treatment with 10 *μ*M DPTA NONOate for 14 days potentiated the migration of the cells approximately 2.5-fold as compared with the nontreated cells, as shown in Figure 2(b).

**Figure 3:**
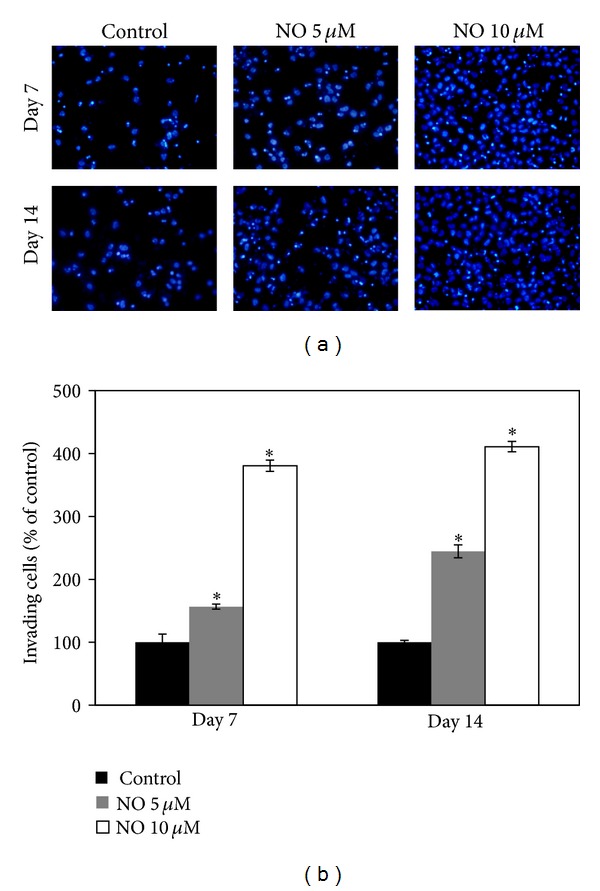
Effect of nitric oxide on H460 cell invasion. The invasion assay was performed using a Boyden chamber. (a) The cells that invaded the underside of the membrane were stained with 10 *μ*g/mL Hoechst 33342 for 10 min and visualized using a fluorescence microscope. (b) The relative cell invasion was determined as described in Materials and Methods. The data are the mean ± SD (*n* = 3). *P < 0.05 versus the control cells.

 In addition, we investigated the effect of NO on H460 cell invasion using a precoated Matrigel Transwell unit, and we found that treatment with the NO donor at various concentrations (0, 5, and 10 *μ*M) for the indicated times significantly stimulated H460 cell invasion through the Matrigel, as shown in Figures 3(a) and 3(b).

### 3.3. NO Enhances Filopodia Formation in Lung Cancer Cells

**Figure 4:**
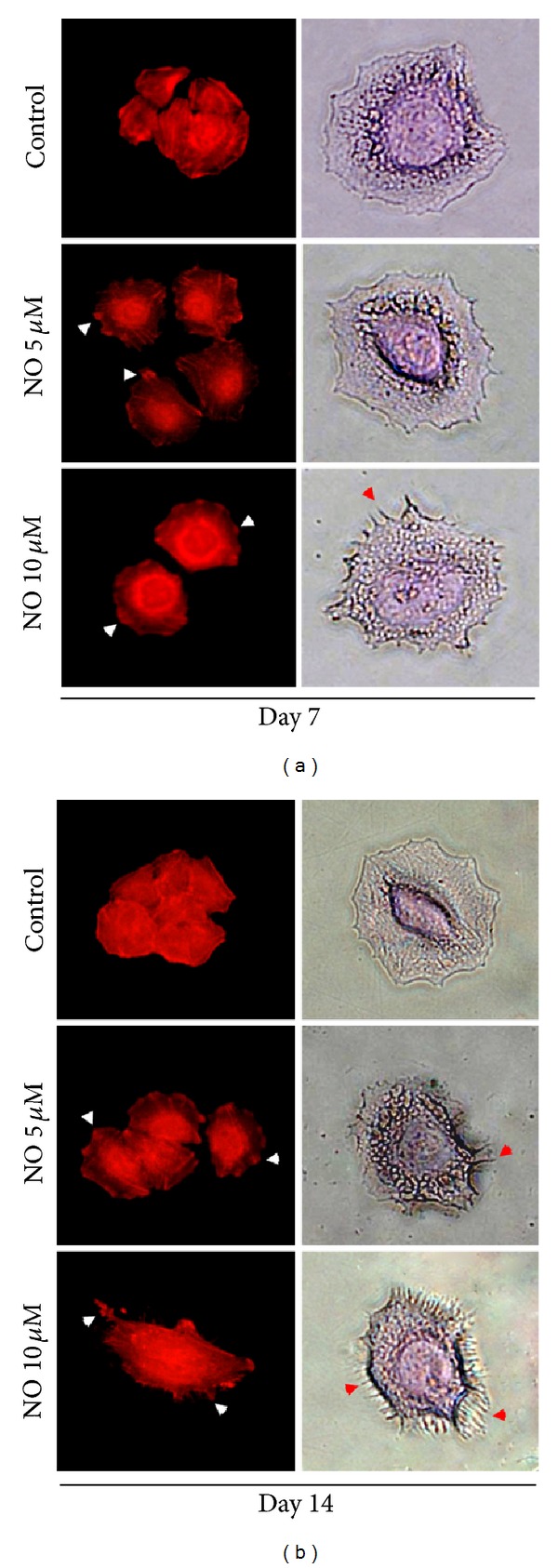
Filopodia formation in H460 cells treated with nitric oxide. H460 cells were treated with NO donor at concentrations of 0–10 *μ*M for (a) 7 days and (b) 14 days. The cells were then stained with phalloidin-rhodamine and sulforhodamine B dye.

Filopodia are generated through actin polymerization and rearrangement of actin filaments, and the formation of filopodia has been linked to increased tumor cell migration. To evaluate the effect of NO treatment on filopodia formation, cells were exposed to NO as previously described, and the presence of filopodia was determined using a phalloidin-rhodamine staining assay. In addition to this staining, the cytoskeletal actin was also stained with sulforhodamine B dye. Figures 4(a) and 4(b) indicate that, when H460 cells were cultured in the presence of the NO donor, the cells exhibited an altered actin alignment and an increased number of filopodia.

### 3.4. The Long-Term NO Exposure Induces Cav-1-Dependent FAK and Akt Activation

**Figure 5:**
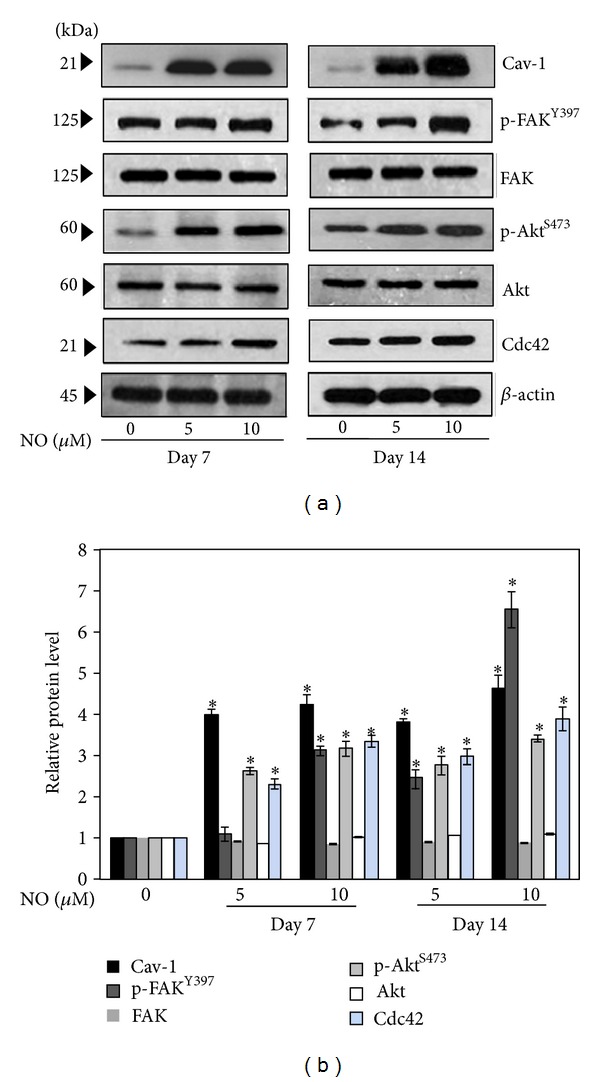
Nitric oxide exposure activates the FAK-Akt pathways. (a) NO-treated cells at 7 and 14 days were subjected to Western blotting, and the expression levels of phosphorylated FAK, total FAK, phosphorylated Akt, total Akt, Cdc42, and Cav-1 were determined. To confirm equal loading of the samples, the blots were reprobed with *β*-actin antibody. (b) The immunoblot signals were quantified by densitometry. The data are the mean ± SD (*n* = 3). *P < 0.05 versus the nontreated control.

Having demonstrated the potentiating effect of NO exposure on lung cancer cell motility, we next examined the underlying mechanism, focusing on the expression levels of the proteins known to play roles in cell migration. Cancer cells were treated with NO donor at different concentrations for 7 and 14 days and were analyzed by Western blotting. Expression levels of the migration-related proteins, namely, Cav-1, FAK, Akt, and Cdc42, were evaluated. Figures 5(a) and 5(b) show that NO exposure for 7 and 14 days significantly increased the levels of Cav-1, phosphorylated FAK (Tyr 397), phosphorylated Akt (Ser 473), and Cdc42, whereas NO exposure had no significant effect on the levels of total FAK and total Akt. Interestingly, the effects of NO on the mentioned proteins appeared to be dose- and time-dependent; cells treated with 10 *μ*M NO donor for 14 days exhibited the most pronounced changes in protein levels as compared to cells treated with 5 *μ*M NO donor or cells that were treated for a shorter period of time. 

**Figure 6:**
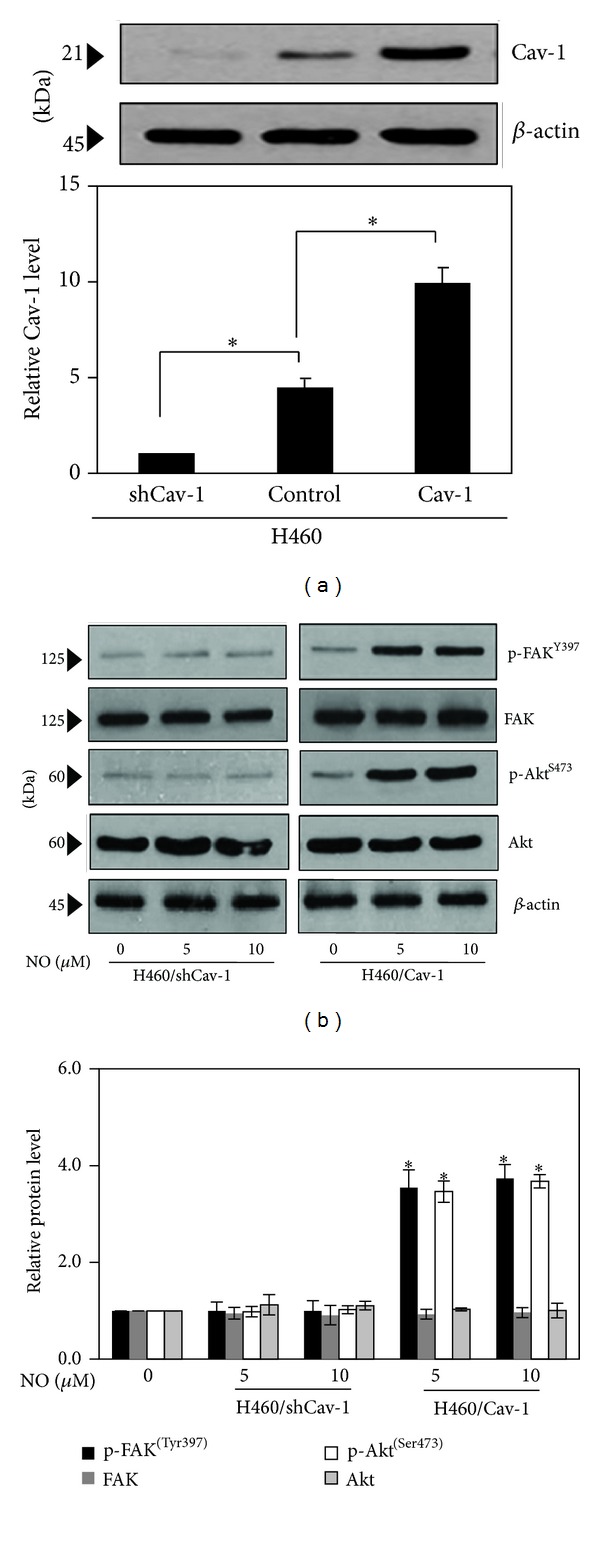
Nitric oxide mediated FAK-Akt activation via Cav-1-dependent mechanism. Stable Cav-1 overexpressed (H460/Cav-1) and Cav-1 knockdown (H460/ShCav-1) cell lines were established as indicated in Materials and Methods. (a) The expression level of Cav-1 protein in the control H460, H460/Cav-1, and H460/ShCav-1 cells was determined by Western blotting. (b) The cells were exposed to NO donor for 14 days, and the expression levels of phosphorylated FAK, total FAK, phosphorylated Akt, and total Akt were determined. The immunoblot signals were quantified by densitometry, and the mean data from the independent experiments were normalized to the control. The data are the mean ± SD (*n* = 3). *P < 0.05 versus the control.

As Cav-1 has been shown to function as an adaptor protein that regulates the activities of other proteins as previously described [21], we tested whether the upregulation of the proteins mentioned previously was through Cav-1-dependent mechanism. Using gene manipulation approaches, Cav-1 overexpressed and knockdown cells were generated as described in Materials and Methods. As expected, Western blot analysis of Cav-1 expression showed a substantial increase in Cav-1 protein level in the Cav-1-transfected cells, whereas a significant decrease in Cav-1 level was observed in the shRNA-Cav-1-transfected cells as compared with the control-transfected cells (Figure 6(a)). The Cav-1 overexpressing cells (H460/Cav-1), the Cav-1 knockdown cells (H460/ShCav-1), and the control H460 cells were cultured in the presence or absence of NO (5–10 *μ*M) for 14 days, and the levels of phosphorylated FAK (Tyr 397), phosphorylated Akt (Ser 473), and their total protein levels were determined.  Figure 6(b) shows that the Cav-1 overexpressed cells (H460/Cav-1) exhibited a significantly increased level of phosphorylated FAK and phosphorylated Akt, whereas the total FAK and total Akt levels were not affected. In contrast, the NO-mediated FAK and Akt phosphorylation events were suppressed in the cells in which Cav-1 was knocked down (H460/ShCav-1 cells). These results indicate that long-term NO exposure in H460 cells induces FAK and Akt activation in a Cav-1-dependent manner. 

## 4. Discussion 

Worldwide, lung cancer is a leading cause of cancer-related death in both men and women [30], and approximately 90% of non-small-cell lung cancer deaths are attributed to cancer metastasis [28, 31]. Among the multiple steps of metastasis, migration of the cancer cells has been recognized as an important hallmark for the successful spread of cancer throughout the body [8, 9]. However, information regarding the key mediators that control the migratory activities of the cancer cells remains largely unknown. An increase in NO production has frequently been observed in the tissue surrounding the tumor and may be critical for some cancer cells behavior [3–7]. In addition, elevated NO production has been observed in the lung tissue of lung cancer patients in comparison with that of normal subjects [6, 7]. These findings have strengthened the idea that NO present in the lung cancer environment may affect the behavior of cancer cells. 

NO is a gaseous molecule that is able to diffuse deeply into tissues; indeed, such a substance has been shown to regulate cell behaviors in many ways, including the relaxation of vascular smooth muscle [3, 32]. Controversial roles of NO have been reported for normal cell motility. NO was shown to inhibit vascular smooth muscle cell migration [32, 33]; however, the opposite effect was observed in the microglia cell model [32, 34]. Accordingly, both the inhibitory effect and promoting effect of NO on cancer cells have been reported [34, 35]. The variable effects of NO in tumors may depend on the localization of NO synthase and its activity, the concentration and duration of NO exposure, and the cellular sensitivity to NO [3–5, 32, 34, 36]. While the long-term effects of NO on lung cancer cell migration are still unknown, Hickok et al. showed that short-term treatment with an NO donor for 4, 6, and 24 h inhibited breast cancer cell migration through N-Myc downstream-regulated gene-1 (NDRG1) expression [35, 37]. However, in prostate cancer cells, NO was shown to potentiate cell motility [37, 38]. The present study demonstrated the novel role of long-term NO exposure in the regulation of lung cancer cell migration that may be important for the fulfillment of cancer insights. Long-term exposure to NO enhances the cells motility via FAK- and Akt-dependent mechanisms. In addition, we provided evidence indicating that such an activation of the FAK-Akt pathway is dependent on the level of cellular Cav-1 (Figure 6). 

Previous studies found that the phosphorylation of FAK at position Tyr 397 is critical for cell migration [12, 13]. Furthermore, FAK action on cell motility was shown to be involved with its downstream Akt [14, 39]. Our gene manipulation experiments further revealed the role of Cav-1 on FAK-Akt pathway. We found that phosphorylated FAK, as well as phosphorylated Akt, increased in response to long-term NO treatment of lung cell lines, and this response was limited in the Cav-1 knockdown cells. However, the upregulation of both phosphorylation events was shown to be intensified in the Cav-1 overexpressed cells (Figure 6). These findings suggest that Cav-1 may have a novel influence on FAK-Akt-mediated cell migration in lung cancer cell models. Cav-1 is the principal component of caveolae membranes. Cav-1 has been reported to promote tumor cell migration and invasion, and an increase in Cav-1 expression is associated with tumor metastasis in lung cancer [17–27]. Consistent with its pro-survival role, Cav-1 positively regulated the growth of lung cancer H460 cells when these cells were treated with NO, as previously described [18]. Since an upregulation of NO, as well as Cav-1 protein, is associated with an aggressive status in lung cancer cells, therefore the results from this study may lead to a better understanding of lung cancer pathology.

Likewise, the small GTPase Cdc42 was shown to regulate actin filaments and the migration of tumor cells [10, 11]. In fibroblasts, Cdc42 induces the rapid formation and extension of filopodia, which are required for movement processes [11, 15, 16]. We investigated how NO exposure affected the actin organization in lung cancer cells and found that NO upregulates Cdc42 protein and enhances the formation of filopodia in these cells. It is worth noting that we did not see a significant change in the level of Cdc42 following ectopic Cav-1 expression (data not shown), suggesting that the NO-mediated Cdc42 increase in this study was through a Cav-1-independent mechanism. Although further investigations may be needed to examine the underlying mechanisms by which NO controls Cdc42 and filopodia formation, this study first revealed the novel effect NO has on cancer cell migration through a Cdc42-dependent mechanism.

## 5. Conclusion

We demonstrated the possible role of long-term NO exposure on the metastatic behaviors of cancer cells, including migration and invasion. NO exposure activated the FAK-Akt signaling pathway through a Cav-1-dependent mechanism and increased filopodia formation. Elevated NO levels have been observed in cancer environments; thus the knowledge gained from the present study may benefit our understanding of cancer biology and may be useful in the development of cancer therapies.

## Abbreviations

Akt: ATP-dependent tyrosine kinase
Cav-1: Caveolin-1
Cdc42: Cell division cycle 42
FAK: Focal-adhesion kinase
NO: Nitric oxide
MTT: 3-(4,5-Dimethylthiazol-2-yl)-2,5-diphenyltetrazolium bromide
p-Akt: Phosphorylated Akt
PBS: Phosphate-buffered saline
p-FAK: Phosphorylated-FAK
TBST: Tris-buffered saline with 0.1% Tween.
